# Omics advancements towards exploring arsenic toxicity and tolerance in plants: a review

**DOI:** 10.1007/s00425-025-04646-9

**Published:** 2025-03-05

**Authors:** Sayyeda Hira Hassan, Melissa Simiele, Gabriella Stefania Scippa, Domenico Morabito, Dalila Trupiano

**Affiliations:** 1 Department of Biosciences and Territory, University of Molise, Pesche, IS Italy; 2 University of Orleans, LBLGC EA 1207, INRAe-USC1328, Orleans, France

**Keywords:** Arsenic toxicity in plants, Omics technologies, Plant response to arsenate/arsenite, Plant stress response

## Abstract

**Main conclusion:**

Omics approaches provide comprehensive insights into plant arsenic stress responses, setting the stage for engineering arsenic-tolerant crops.

**Abstract:**

Understanding arsenic (As) toxicity in plants is crucial for environmental and agricultural sustainability, considering the implications of As in impacting soil productivity and environmental health. Although some articles already examined the detailed molecular mechanisms behind As toxicity and tolerance, a comprehensive review of recent omics advancements in studying plant responses to As exposure is needed. The present review highlights the valuable contribution of omics approaches (genomics, transcriptomics, proteomics, and metabolomics) to characterize the intricate response to As overall, which could empower As-tolerant plant development. Genomic techniques, such as QTL mapping, GWAS, RAPD, and SSH, hold the potential to provide valuable insights into the genetic diversity and expression patterns associated with the plant response to As stress, highlighting also the power of new advanced technology such as CRISPR–Cas9. Transcriptomics approaches (e.g., microarrays and RNA sequencing) revealed gene expression patterns in plants under As stress, emphasizing the role of sulfur metabolism in As tolerance. Proteomics, using 2-DE combined with MALDI-ToF MS or ESI–MS/MS, offers insights into the stress-inducible proteins and their involvement in As toxicity mitigation, while iTRAQ-based proteomics enabled an understanding of cultivar-specific responses under high As concentration. Metabolomics, with LC–MS, GC–MS, (U)HPLC, and NMR, elucidated small molecule alterations and complex metabolic activities occurring under As plant exposure. Compendium of data and evidence-related tools offers a foundation for advancing As-tolerant plant development and promoting environmental and agricultural resilience.

## Introduction

Intense human activities and modern agricultural practices have led to widespread concern about the contamination of the environment with toxic metals which can be detrimental to the ecosystem (Rai et al. 2019; Gashi et al. 2020). Non-essential metal(loid)s, such as arsenic (As), lead (Pb), cadmium (Cd), and mercury (Hg), are toxic to plants even at low concentrations (Raza et al. 2021; Mawia et al. 2021). Among these, As is the 20th most abundant mineral naturally developed on earth’s crust and is widely spread across the land, air, and water (Cullen and Reimer 1989). Also, As is ranked at the top of the hazardous substance priority list by the Agency for Toxic Substances and Disease Registry (ATSDR 2023). The major threat to public health lies in its contamination of groundwater. Furthermore, As is commonly found in plant-based diets, including cereals, vegetables, and fruits (Tripathi et al. 2007).

Naturally, As exists in both inorganic and organic forms. The inorganic species, including arsenite [As(III)] and arsenate [As(V)], are more abundant in terrestrial ecosystems. As(V) is more predominant under aerobic soil conditions; As(III) is more frequent in anaerobic conditions, such as flooded rice fields (Shri et al. 2019; Tang and Zhao 2021). On the other hand, organic As species are present at relatively low levels in soil, mainly due to the historical use of As-based agrochemicals in agricultural fields and the influence of microbial activity (Al-Makishah et al. 2020; Huang et al. 2011). However, some organic species of As, especially the methylated species—monomethylarsonic acid (MMA), dimethylarsinic acid (DMA), and trimethylarsine oxide (TMAO)—are commonly abundant in soil (Finnegan and Chen 2012). As speciation greatly influences its uptake by plants. As(III) enters the plant through aquaglyceroporins, while As(V) is generally taken up by phosphate transporters (PHTs) because of its chemical resemblance with phosphate (Li et al. 2016). Other transporters involved in As uptake and translocation include C-type ATP-binding cassette (ABCC), natural resistance-associated macrophage protein (NRAMP) transporters, multidrug and toxic compound extrusion (MATE) transporters, inositol transporters, As compounds resistance (ACR), and auxin transporters (PIN-FORMED or PIN) (Mondal et al. 2022).

As exerts its toxic effect by interfering with various metabolic processes, and the mechanisms that plants use to respond are not simple detoxification actions. The As-stress response is complex and regulated by different signaling pathways that lead to the activation or suppression of specific genes (Mondal et al. 2022). Several signaling pathways are activated upon As exposure, including those related to reactive oxygen species (ROS)/oxidative stress, mitogen-activated protein kinase (MAPK), calcium, and phytohormones. Due to its capacity to promote the generation of ROS in cells, As has been classified as an external oxidative stressor by inhibiting the key enzyme systems along with electron leakage during the conversion of As(V) to As(III). ROS have the potential to significantly oxidatively damage proteins, and DNA in cells (Lin et al. 2008), and further stimulate the peroxidation of polyunsaturated fatty acids of the cell membrane lipid bilayer which leads to an increase in electrolyte leakage, H_2_O_2_ content, and amount of thiobarbituric acid-reacting substances (Chakrabarty et al. 2009). The As-induced oxidative stress can lead to a reduction in soluble protein content, glutathione, photosynthesis rate, and pigment depletion (Chandrakar et al. 2016) and downregulation in several genes involved in cell growth, cell cycle, and morphogenesis(Norton et al. 2008). In rice, Chauhan et al. (2020) revealed that oxidative stress due to As elevated the expression of stress proteins involved in ROS homeostasis and defence mechanism, i.e., L-ascorbate peroxidase 8 (Os-0609), L-ascorbate peroxidase 2 (Os-8615), glutathione S-transferase (Os-7221), heat shock protein 70 (Os-1817), and lactoylglutathione lyase (Os-4832). Moreover, analytical analysis of the transcriptome of *Arabidopsis* illuminates the complex dynamics: genes related to the biogenesis of secondary cell walls, cell cycle regulation, and oligopeptide transport are primarily downregulated in response to As(V)-induced stress, whereas signaling pathway genes, such as MAPK, MAPK kinase, and Ca^2+^-dependent protein kinase (CDPK), show upregulation (Huang et al. 2012).

**Fig. 1:**
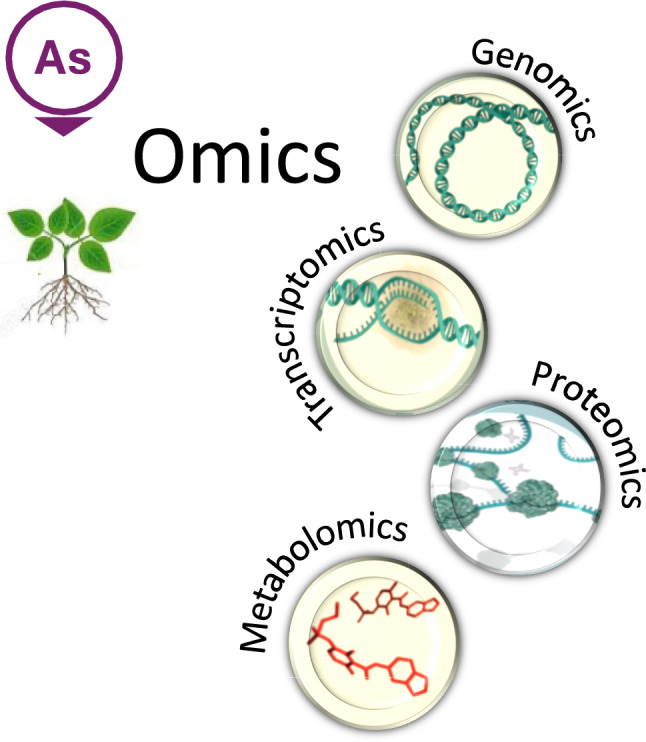
“Omics” approaches to understand the arsenic-induced toxicity and tolerance in plants

Genes, RNA, metabolites, proteins, and other molecular regulators, along with associated processes like transcription, translation, post-translation, replication, and so on, are essential to the execution and upkeep of many essential plant functions. However, the genetic regulatory mechanism are not completely understood (Raza et al. 2022). In recent years, several “Omics” approaches, including genomics, transcriptomics, proteomics, and metabolomics (Fig. 1), have been implied to understand the As-induced toxicity and tolerance in plants (Khan et al. 2021; Jamla et al. 2021). The surge in omics research reflects the growing demand for comprehensive insights into the molecular mechanisms of plant responses to environmental stressors like As. Even though there are articles reviewing the detailed molecular mechanisms behind As toxicity and tolerance in plants, a comprehensive review compiling the recent advancements towards the molecular changes in plants after being exposed to As is currently lacking. Thus, the presents review intends to highlight the recent studies about As plant exposure using state-of-the-art *Omics* approaches (genomics, transcriptomics, proteomics, and metabolomics) indexed in Google Scholar (https://scholar.google.com/) and PubMed (https://pubmed.ncbi.nlm.nih.gov/) databases from 2005 to 2024 (Table 1). Targeted keywords (“Genomics”; “Transcriptomics”; “Proteomics”; Metabolomics”; “As stress in plants”) were used to filter research articles that had the defined search phrase in the title, abstract, introduction, or keywords. Furthermore, to identify the most prevalently utilized technologies, the search included terms specific to the study of As impact on plants, such as “arsenic stress genomics in plants”, “transcriptomics arsenic response in plants”, “proteomics arsenic response in plants”, and “metabolomics arsenic response in plants”.

**Table 1:** Literature review relevant to “omics” techniques to understand arsenic (As)-induced changes in different plant species

Plant species	Techniques used	Arsenic species	Response to arsenic exposure	References
*Zea mays* L	QTL mapping	–	11 QTLs associated with As concentration in leaves, stems, bracts and Kernels	Ding et al. 2011
*Zea mays* L		–	28 QTLs related to arsenic concentration across two different location (14 QTLs in each)	Fu et al. 2016
*Oryza sativa *L		As(V)	Four As phytotoxicity-related QTLs were identified with significant one on chromosome 8	Syed et al. 2016
*Oryza sativa *L		–	Three significant QTLs accounted for 73% of the phenotypic variance in grain dimethylarsinic acid (DMA) concentration	Kuramata et al. 2013
*Oryza sativa *L		–	2 QTLs were identified in root and shoot (one for each) at seedling stage, and 2 QTLs an maturity stage	Zhang et al. 2008
*Oryza sativa *L	RAPD	As(III)	As (III)-induced genomic damage in two rice varieties	Ahmed et al. 2012
Wheat (*Triticum aestivum*)		As(III)	A dose-dependent increase in genomic alteration upon As exposure	Aksakal and Esim 2015
*Crambe abyssinica* (Abyssinian mustard)	PCR-Select Suppression Subtraction Hybridization (SSH)	As(V)	Identified 38 different genes from 105 differentially expressed subtracted cDNAs	Paulose et al. 2010
*Panax notoginseng*	RNA-seq	As(V)	1725 DEGs were identified encoding hormone signaling, As hyperaccumulation, oxidative stress	Liu et al. 2016
*Oryza sativa *L		As(III)	DEGs identified under arsenite stress, primarily associated with transmembrane transport and ion binding	Xiao et al. 2021
*Brassica juncea*		As(V)	Identification of DEGs involved in arsenic reduction, phytochelatin production, and arsenic sequestration in vacuoles, as well as genes related to reactive oxygen species (ROS) production and detoxification mechanisms	Yanitch et al. 2017
*Oryza sativa* indica DOURADOAGULHA		As(III) and As(V)	2983 and 1844 genes were identified responsive to As(III) and As(V) stresses, respectively	Di et al. 2021
Rice (Indica variety, Minghui 86)	RNA Seq, MALDI-TOF/TOF	As(III)	Important metabolic pathways and transporters that control the interplay between As and selenium, reducing As buildup and enhancing plant growth	Chauhan et al. 2020
*Brassica juncea*	Microarray	As(V)	A total of 1,285 DEGs upon As(V) exposure related to various signaling pathways including hormones and kinases	Srivastava et al. 2015
*Oryza sativa *L		As(III) and As(V)	Identification of DE miRNAs in contrasting arsenic accumulating cultivars in response to As(III) and As(V) stress	Sharma et al. 2015
*Arabidopsis thaliana*		As(V)	Identified 168 and 548 DEGs in the most tolerant (Col-0) and sensitive (Slavi-1) accessions, respectively, with 120 common DEGs	Shukla et al. 2018
*Oryza sativa *L		As(III) and As(V)	Identification of 171 and 428 DEGss in response to exposure to AsIII and AsV, respectively	Chakrabarty et al. 2009
*Oryza sativa *L		As (V)	11 class I metallothionein (MT) genes in the rice genome	Gautam et al. 2012
*Oryza sativa *L		As(V)	A total of 576 probe sets were upregulated and 622 were downregulated in response to arsenate exposure	Norton et al. 2008
*Oryza sativa *L		As(V)	Induction of genes involved in abiotic stress response, detoxification pathways, and secondary metabolic processes, and downregulation of genes associated with secondary cell wall biogenesis, cell cycle, and oligopeptide transport	Huang et al. 2012
*Oryza sativa *L		As(V)	Induction of genes involved in oxidative stress response, detoxification pathways, and secondary metabolic processes	Dubey et al. 2014
*Brassica juncea*		As(V)	A total of 69 miRNAs were identified related to developmental processes, sulfur uptake, transport, assimilation, and hormonal biosynthesis/function	Srivastava et al. 2013
*Arabidopsis thaliana*		As(III)	Identification of putative targets of SLIM1 (sulfur limitation 1) and role of SLIM1-TF in As-sensitivity	Jobe et al. 2021
*Arabidopsis thaliana*	Whole genome oligonucleotide microarrays	As(V)	Highlighted induction of antioxidant-related genes, and the repression of genes typically induced by phosphate starvation	Abercrombie et al. 2008
*Oryza sativa* L., ssp. Indica	Deep sequencing	As(III)	Identified 67 As(III)-responsive miRNAs in indica rice roots	Liu and Zhang 2012
*Zea mays* L		As(V)	22 upregulated and 35 downregulated As(V)-responsive miRNAs	Ghosh et al. 2017
*Oryza sativa *L	Illumina sequencing	As(III)	Identified genes involved in heavy metal transportation, jasmonate biosynthesis and signaling, and lipid metabolism, 36 new As(III)-responsive miRNAs	Yu et al. 2012
*Oryza sativa *L	2-DE, MALDI-TOF–MS	As(V)	23As-regulated proteins in root were observed, S-adenosylmethionine synthetase, oxophytodienoic acid reductase, and glutathione S-transferases were upregulated, while proteins like protein disulfide isomerase and glutamine synthetase root isozyme were downregulated	Ahsan et al. 2008
*Oryza sativa *“Nipponbare”		As(V)	Identification of 38 DEPs involved involved in redox homeostasis in shoot	Liu et al. 2013
*Spinacia oleracea*		As(V)	Positive role of sulfur in mitigation of As-induced oxidative stress	Amna et al. 2020
*Zea mays* L		As(III) and As(V)	Revealed proteome related to oxidative defense system in root	Requejo and Tena 2005
*Zea mays* L		As(III) and As(V)	74 DEPs, including key enzymes and regulatory proteins in shoot	Requejo and Tena 2006
*Artemisia annua*		As(V)	Increased photosynthetic CO2 assimilation and usage of carbon resources under As(V) stress	Rai et al.(2014
*Artemisia annua*		–	synergistic effect of salicyclic acid in modulating the plant's tolerance to As stress	Kumari et al. 2018
*Oryza sativa* L	2-DE, MALDI-TOF/TOF	As(III)	selenium supplementation reversed the As-induced structural changes by suppressing As-transporting genes and sulfate transporter genes	Chauhan et al. 2020
*Oryza sativa *L		As(III)	Total of 113 DEP were identified by 2-DE gel and 80 confirmed by MALDI-TOF which were involved in glycolysis, TCA cycle, AA biosynthesis, protein metabolism, photosynthesis, stress, and energy metabolism under sulfur alleviated arsenic toxicity	Dixit et al. 2015
*Oryza sativa* L. cv. Dongjin	2-DE, MALDI-TOF MS, ESI–MS/MS	As(V)	Energy metabolism and photosynthetic activity were altered under As(V) exposure	Ahsan et al. 2010
*Pteris vittata*	2-DE, nano-LC-Q-TOF MS/MS	As(V)	Protective effect of arbuscular mycorrhiza symbiosis toward As stress	Bona et al. 2011
*Pteris vittata*	2-DE, LC–MS/MS	As(V)	Two key enzymes, Pv2.5–8 and PvACR2 were identified, involved in reducing arsenate to a less toxic form in both sporophytes and gametophytes	Cesaro et al. (2015)
*Pteris vittata*		As(V)	Colonization by Glomus mosseae and Gigaspora margarita differentially modulated the expression of 130 leaf proteins in response to As, including As transporter, PgPOR29 as upregulated protein	Bona et al. 2010
*Populus*	2-DE, MALDI-TOF/TOF MS	As(V)	Protein PdDRT102 was highly induced in the arsenate-tolerant Populus cv.‘zhonglin 2025’	Liu et al. 2017
*Agrostis tenuis*		As(III) and As(V)	arsenic exposure, particularly As(III), led to the disruption of photosynthesis and induced changes in proteins associated with defense mechanisms, such as cysteine protease inhibitors	Duquesnoy et al. 2009
*Brassica napus*	iTRAQ labeling, RPLC–MS/MS	As(III)	Highlighted cultivar-specific responses, with cultivar ZS758 showing better tolerance to arsenic stress compared to ZD622, as evidenced by the distinct protein expression patterns	Farooq et al. 2021
*Brassica napus*		As(III)	Highlighted the role of methyl jasmonate's role in mitigating As-induced oxidative stress	Farooq et al. 2018
*Oryza sativa* L	HPLC–ICP-MS/ESI–MS	As(V)	identification and quantification of phytochelatins in rice roots exposed to arsenic	Lemos et al. 2014
*Oryza sativa* L	LC–MS	As(V)	Major metabolite alterations were found in glycerolipids, glycerophospholipids, and amino acid-related pathways	Pérez-Cova et al. 2022
*Arabidopsis*		As(V)	Alteration in antioxidative enzymes, nitric oxide (NO) and S-nitrosoglutathione (GSNO) metabolism	Leterrier et al. 2012
*Oryza sativa* L	HPLC, ICPMS	As(III)	Increased thiol metabolism and amino acid content in tolerant variety	Tripathi et al. 2013
*Oryza sativa* L		As(V)	underlines the role of thiol-mediated detoxification mechanisms in conferring arsenic tolerance	Tripathi et al. 2012
*Oryza sativa* L	UHPLC	As(V)	Identified approximately 4000 metabolites that were up- and downregulated in rice seedlings under As stress	Yadav et al. 2021
*Andrographis paniculata*	HPLC	As(V)	Increase in the flavonoids under As stress	Das et al. 2021
*Oryza sativa* L	HPLC, MALDI-TOF-TOF	As(III)	Histidine was remarkably increased under As(III) and high sulfur supplementation, while valine significantly decreased under low sulfur supplementation with As(III) treatment	Dixit et al. 2015
*Cucumis sativus* L	RP-HPLC, ESI–MS	As(V)	Identified changes in five metabolites, with isoleucine being notably downregulated	Uroic et al. 2012
*Salvadora persica* L	GC–MS, HPLC	Sodium arsenate	64 metabolites were identified under salinity and/or As stress, and early As-salinity stress tolerance-related metabolites	Patel and Parida 2022
*Pteris cretica* and *Spinacia oleracea*		As(V)	In P. cretica, the total content of free amino acids were decreased significantly, while in S. oleracea, the content of these amino acids increased	Zemanová et al. 2021
*Brassica juncea*		As(V)	As(V) exposure induce four brassinosteroids (teasterone, castasterone, typhasterol and 24-epibrassinolide) in Brassica juncea	Kanwar and Bhardwaj, 2015)
*Mentha arvensis* L		As(V)	Reduction in menthol content by As(V) at 100 mg As kg-1 of soil)	Nabi et al. 2019
P*teris vittata, Pteris multifida, Pteris semipinnata*		As(V)	Organic acid concentration changes in ferns is not directly correlate with As accumulation	Wang et al. 2011
Microalga *Scenedesmus*	NMR		45 different metabolites were identified, including amino acids, organic acids, nucleotides, sugars, osmolytes, and phosphagens	Arora et al. 2018

## Genomics

**Fig. 2:**
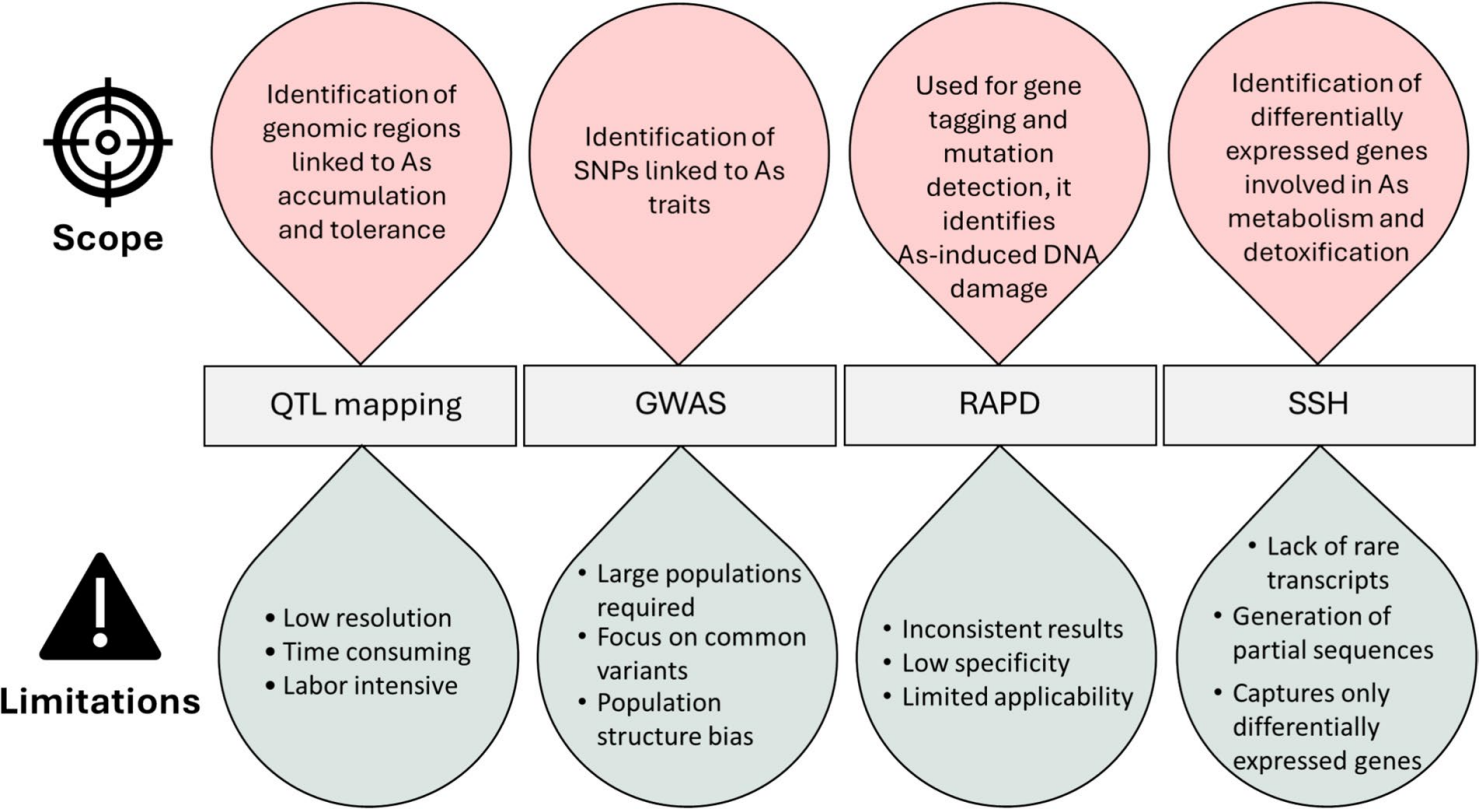
Scope and limitations of genomic approaches—quantitative trait loci (QTL) mapping, genome-wide association studies (GWAS), random amplified polymorphic DNA (RAPD), and PCR-select suppression subtraction hybridization (SSH)—in understanding the arsenic (As)-induced toxicity and tolerance in plants

At the molecular level, stress response is controlled by a collection of genes and a variety of mechanisms ranging from transport to metabolism. This complex network is essential, as it helps plants to adapt and survive under stress conditions. Therefore, employing a genomics approach to sequence and annotate these genes is beneficial for enhancing phytoremediation purposes (Sahoo et al. 2020). A comprehensive study of all genes (whole genome), their function, structure, evolution and mapping is crucial, unlike genetics focuses on a single gene or variant. Understanding genetic variation can be greatly aided by genomics, which could improve plant breeding effectiveness and lead to genetic advancement (Yang et al. 2021; Kaur et al. 2021). Genomic approaches, including quantitative trait loci (QTL) mapping, genome-wide association studies (GWAS), random amplified polymorphic DNA (RAPD), and PCR-select suppression subtraction hybridization (SSH) (Fig. 2), have been employed to gain valuable insight towards exploring the genetic base of plant responses to As stress.

**Fig. 3:**
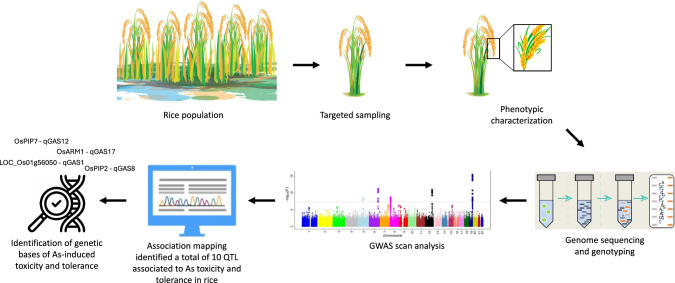
Genome-wide association studies (GWAS) workflow in understanding the arsenic (As)-induced toxicity and tolerance in rice

In this regard, QTL mapping serves as a foundational technique to identify and locate the genomic regions associated with the variation of complex traits in plants. This technique is particularly useful in understanding the genetic basis of traits that are influenced by certain environmental factors and multiple genes (Sehgal et al. 2016). Several studies used mapping to identify QTLs associated with As toxicity and tolerance. Ding et al. (2011) analyzed different tissues of maize (*Zea mays* L.)—leaves, stems, bracts, and kernels—by utilizing recombinant inbred lines (RILs) derived from an elite maize hybrid, Nongda108. A total of 11 QTLs associated with specific markers in the different tissue, mainly due to the diverse accumulation: leaves > stems > bracts > kernels. In detail, in leaves, the markers included umc1243 (chromosome 1), umc1229 (chromosome 5), and umc1075 (chromosome 8). In stems, the identified markers were umc1243 (chromosome 1), umc1229 (chromosome 5), and umc1015 (chromosome 9). In bracts, the markers umc1243 (chromosome 1) and umc1015 (chromosome 9) were detected, while in kernels, the markers umc1028 (chromosome 3), umc1229 (chromosome 5), and umc1056 (chromosome 7) were associated with QTLs. Markers umc1243 (chromosome 1) and umc1229 (chromosome 5) were identified across multiple tissues, indicating some consistency in these genomic regions for As concentration regulation. Another study focused on identifying QTLs for As accumulation in maize, using a recombinant inbred maize line population derived from the Chinese crossbred variety (Yuyu22). This study was conducted across two different sites with varying soil As levels to understand the environmental influence on As accumulation. The researchers reported a similar trend in As distribution, with maize kernels exhibiting lower As concentration compared to other tissues such as axes, stems, bracts, and leaves. A total of 28 QTLs (14 QTLs at each location) related to As concentration were identified (Fu et al. 2016). In contrast to these studies that focused on As accumulation-related QTLs, Syed et al. (2016) studied As tolerance-related QTLs. To do this, selective genotyping utilizing 98 microsatellite markers was employed to map phytotoxicity tolerance-related QTLs during the seeding stage of an F2 population derived from a cross between an As-tolerant indica rice cultivar (BRRI dhan47) and As-sensitive indica rice cultivar (BRRI dhan45). Four QTLs were identified as associated with relative growth in shoot and root length, and shoot–root biomass with the most significant As phytotoxicity tolerance-related QTL on chromosome 8 (Syed et al. 2016). Using 704 single nucleotide polymorphisms (SNPs), a marker–trait association study was carried out for As-linked traits. With phenotypic variances ranging from 8.6 to 12.6%, nine QTLs were found. Six of these QTLs for shoot As content were mapped on chromosomes 2, 5, 6, and 9, two of the QTLs in roots were mapped on chromosome 8, and one QTL for relative Chl content was mapped on chromosome 1. These QTL intervals were used to identify 25 candidate genes that were connected and showed transcription regulation for As toxicity-linked traits (Murugaiyan et al. 2019). Kuramata et al. (2013) conducted a study focusing on the accumulation of DMA, an organic form of As, in rice grains by generating a F2 population from a cross between two contrasting cultivars: Koshihikari, known for low DMA accumulation, and Padi Perak, characterized by high DMA accumulation. Their analysis led to the identification of three QTLs on chromosomes 6 and 8, accounting for approximately 73% of the phenotypic variance in grain DMA concentration. A study suggested a possible shared genetic mechanism between factors controlling As accumulation and phosphorous accumulation while mapping QTLs associated with As accumulation in rice. Four QTLs were detected in a double haploid population derived from a cross between the India (TN1) and the Japonica (CJ06) cultivars. Specifically, one QTL was located on chromosome 2 in the shoot and another on chromosome 3 in the roots at the seedling stage. While two QTLs were identified on chromosomes 6 and 8 at the maturity stage (Zhang et al. 2008). Specifically, QTL on chromosome 8 was found to influence As concentration in rice grain as well as phosphorous concentration in shoot of rice seedlings. However, despite the QTL advantages as a commonly used genomics technique, it can suffer from low genetic resolution besides low throughput, being time-consuming, and labor-intensive, which can hinder its applicability in large-scale studies (Bohra et al. 2020). Addressing these challenges often requires the integration of more advanced genomic approaches, such as GWAS, to build on the insights provided by QTL mapping. GWAS offer improved resolution and the ability to link certain genetic variations with particular attributes in different individuals. It overcomes many limitations of classical trait mapping by providing improved resolution and utilizing trials from previously studied bread wheat and rice populations in which genetic changes can be linked with a phenotypic difference (Raza et al. 2022). Despite this, GWAS often relies on previously studied populations, limiting novel discoveries in unexplored groups. While it identifies loci associated with traits, these often overlap with known regions (Tam et al. 2019). Zhao et al. (2018) performed GWAS on maize plants and identified six single nucleotide polymorphisms (SNPs) linked to four QTLs associated with As accumulation. These loci were situated between 25.71 to 25.77 Mb on chromosome 1, overlapping with previously identified QTLs. Among them, only one candidate gene was found: GRMZM2G130987, which is known to encode a protein involved in P–P bond hydrolysis-driven protein transmembrane transporter activity, playing a role in As transport. Additionally, other SNPs on chromosome 2 were located within the BAsA2/XAsA2 QTL, where five candidate genes were identified, including GRMZM2G125495, which encodes a protein with extracellular glutamate-gated ion channel activity. This research lays a strong foundation for further exploration into how these genes influence phenotypic variation or environmental responses (e.g., As accumulation or transport). As summarized in Fig. 3, Liu et al. (2019) also employed GWAS focusing on the presence of toxic metals in rice grains and identified 10 As-related QTLs. Interestingly, *OsPIP2;7* and *OsARM1* harbored in the identified QTLs (*qGAS8*, *qGAS12*, and *qGAS17*) were reported to be involved in As tolerance and detoxification. Successively, they focused on qGAS1, which exhibited the most significant P-value across the entire rice population, examining whether transporter-related genes could be the candidate gene. Although, *LOC_Os01g56050,* encoding a transport protein belonging to the MATE family, involved in metal detoxification, resulted the best candidate, it need to be further confirmed using CRISPR/Cas9 editing approaches.

Thus, the reliance of GWAS on already known QTLs and candidate genes, and the lack of functional validation, highlights the need for integrative approaches, such as functional genomics and transcriptomics, to provide deeper biological insights (Cano-Gamez and Trynka 2020). While QTL mapping and GWAS help in identifying genetic loci associated with complex traits, techniques like RAPD complement these approaches by focusing on genomic alterations caused by stress conditions. It is one of the PCR-based genomic techniques for molecular markers which can be of great importance for tagging and mapping the gene of interest. These markers are amplified DNA segments using a single primer of arbitrary nucleotide sequence with low annealing conditions (Babu et al. 2021). Beyond its extensive use for the classification of species, mapping and phylogenetic studies, RAPD has shown its possible use in assessing As-induced genomic DNA damage and mutation, serving as a basis for a novel biomarker assay to detect DNA damage. For example, Ahmad et al. (2012) reported As (III)-induced genomic damage by using the RAPD technique utilizing 11 different primers and observed alteration in band patterns between control and As (III)-treated rice varieties (IR64 and PB1). The study revealed alterations in band patterns between control and As(III)-treated samples, with an increase in bands in PB1 and a decrease in IR64, aligning with the physiological results with reduced seed germination and length along with less chlorophyll and protein content with more significant effects in the IR64 variety. In another study, Aksakal and Esim (2015) reported significant changes in the DNA pattern of wheat seedlings treated with different concentrations of arsenic trioxide (As_2_O_3_) by employing the RAPD technique which was indicative of DNA damage or mutations caused by As exposure. These studies demonstrate the effectiveness of RAPD as an investigational tool for assessing As-induced genomic alteration. Despite its utility, the use of RAPD could experience challenges due to its inherent variability and incapability of consistency across different experimental conditions, making it challenging to differentiate between variations caused by As stress and those resulting from experimental inconsistencies (Babu et al. 2021; Zhang et al. 2011).

Complementing RAPD, SSH is another PCR-based technique, effective in differentiating two closely linked DNA samples and identifying the unique sequences present in one set but absent in the other by suppressing the amplification of common cDNAs while amplifying differentially expressed cDNAs (Sahebi et al. 2015; Badapanda 2013). Paulose et al. (2010) employed PCR-Select SSH to identify differentially expressed transcripts and pathways involved in As metabolism and detoxification in plants under As(V) stress. A total of 105 differentially expressed subtracted cDNAs were sequenced, representing 38 genes. These genes encode proteins functioning as metal transporters, antioxidants, reductases, enzymes involved in protein degradation pathways, and several uncharacterized proteins. The transcripts corresponding to these subtracted cDNAs showed strong upregulation under As(V) stress. However, there are drawbacks to employing the SSH-based technique, such as the potential to miss rare transcripts due to its reliance on relative abundance for differential amplification. This limitation restricts its ability to capture low-expression genes that might play critical roles in specific stress pathways. Also, its applicability is restricted to gene expression investigations making it a narrower tool compared to whole-genome sequencing approaches. Moreover, the sequences generated through SSH are often partial-length contigs which can complicate downstream analyses (Sahebi et al. 2015; Badapanda 2013). The results from these studies can be beneficial in breeding varieties with lower As concentrations specifically in edible parts of plants. Moreover, understanding genetic bases can aid in developing varieties for phytoremediation purposes in As-contaminated soil. These studies provide information on genetic insights, but do not address the interaction between these identified genetic factors and environmental conditions, which can significantly influence As toxicity and tolerance. Also, while QTL mapping and GWAS offer the initial stage for comprehending complex traits, and RAPD and SSH provide insights into genetic diversity and differential gene expression, their limitations could hinder the rapid application of findings to breeding programs in order to develop As-resistant crops. In this regard, advanced genomic technologies like CRISPR–Cas9 (clustered regularly interspaced palindromic repeats-associated protein 9) can enable precise gene editing and direct manipulation of genes associated with As tolerance. This approach holds significant promise for enhancing plant resilience to As stress. By targeting specific genes involved in stress responses, this technology facilitates the development of plants better equipped to withstand adverse environmental conditions (Kumar et al. 2023; Asmamaw and Zawdie 2021). Moreover, regardless of the remarkable progress in genomics, a thorough assessment of other omics technologies is necessary to obtain a comprehensive molecular understanding. Also, the genomics approach helps identify whether specific target genes are present or absent in an organism. However, to understand the functional role of these genes, it is crucial to carry out expressional analysis. In this context, transcriptomics becomes the preferred method for revealing the activity and function of these genes (Mehta et al. 2019).

## Transcriptomics

**Fig. 4:**
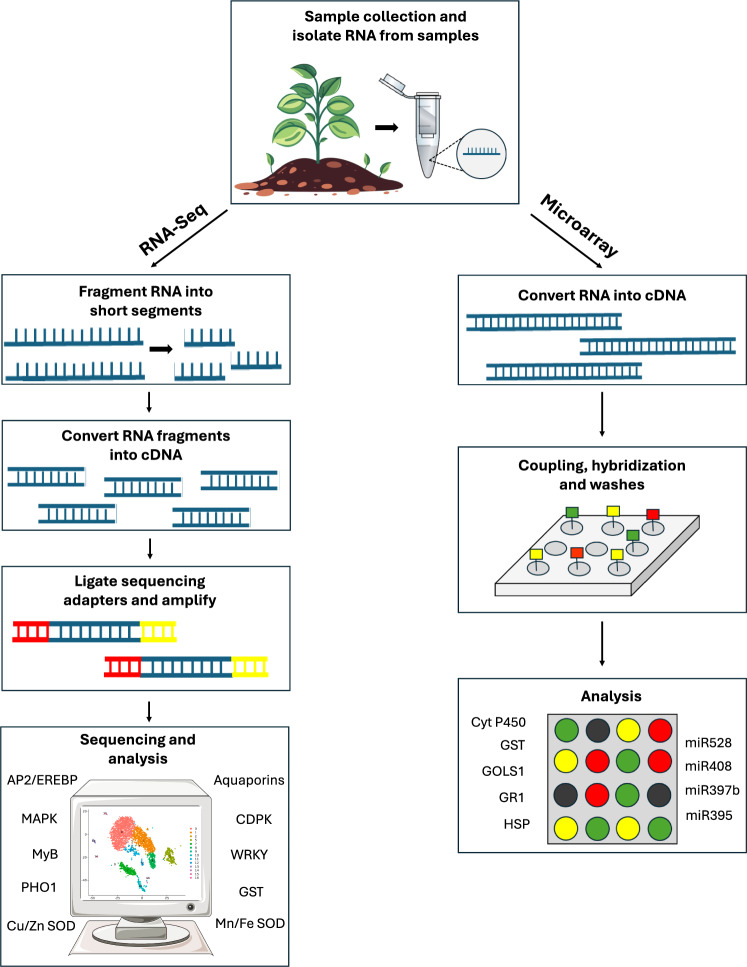
RNA-sequencing (RNA-Seq) and microarray application to study arsenic stress response

Transcriptomics is the study of a complete set of all the ribonucleic acid (RNA) molecules (transcripts) expressed in some given entity, from single cell to the entire organism. The versatility of transcriptomic methods offer new insight on the effects of stress. Indeed, identifying stress-induced/suppressed genes may lead to the discovery of novel proteins, or transcription factors responsible for regulating stress response (Alvarez et al. 2015). At the moment, microarrays and RNA-seq are at the forefront methodologies for high-throughput transcriptome analyses (Rai et al. 2018) leading to the identification of several key factors involved in As stress response and tolerance (Fig. 4).

Several microarray-based transcriptomic studies have established the connection between As stress and oxidative damage. Using this analysis, transcripts of three classes of superoxide dismutase (Cu/Zn SOD, Mn SOD, Fe SOD) were reported to be regulated by As(V) stress in *A. thaliana* (Abercrombie et al. 2008). Also using microarray analysis, Srivastava et al. (2015) identified 1285 differentially expressed genes (DEGs) upon As(V) exposure and key genes related to As-induced oxidative damage in Indian mustard. This study also highlighted As(V) exposure regulated WRKY, Myb, AP2/EREBP, basis helix-loop-helix, G2-like, and DOF zinc finger. However, gene expression patterns upon As-induced oxidative stress can be varied in crop cultivars as reported by Shukla et al. (2018). They analyzed *Arabidopsis* tolerant cultivar (Col-0) and sensitive cultivar (Slavi-1) under As(V) stress using microarray, and found differentially modulated transcriptome with significantly overexpressed genes to detoxify As(V) stress. As-stress-responsive genes encoding GR1, Cyt P450, HSPs, and GSTs were upregulated in the sensitive cultivar. These genes play essential roles in detoxification pathways and mitigating oxidative damage caused by As-induced ROS accumulation. For instance, GSTs facilitate the conjugation of As to glutathione for sequestration (Jain et al. 2010), while HSPs aid in maintaining protein stability under stress (Gupta et al. 2010). Similarly, CYPs are involved in xenobiotic detoxification (Pandian et al. 2020), and GR1 contributes to the regeneration of reduced glutathione (Couto et al. 2016), a crucial antioxidant. In contrast, the tolerant Col-0 exhibited upregulated expression of AtGolS1 and peroxidase genes, suggesting their involvement in enhancing osmoprotection and ROS scavenging under As stress. AtGolS1 is implicated in the synthesis of galactinol, a critical osmoprotectant, protecting cells against oxidative damage (Nishizawa et al. 2008). Meanwhile, peroxidases play a pivotal role in reducing hydrogen peroxide levels, thereby preventing cellular damage caused by oxidative stress (Pandey et al. 2017). By employing microarray, Sharma et al. (2015) analyzed the role of miRNAs in the contrasting response of high As accumulating rice (HARG) and low As accumulating rice (LARG) and found that a set of miRNAs from different families were differentially expressed in both cultivars in response to As(III) and arsenate As(V) stress. The majority of miRNAs were downregulated in both HARG and LARG, suggesting the modulated expression of miRNA as a key factor in determining the variable responses of pant cultivars to As stress.

Combining microarray analysis with the DNA Affinity Purification sequencing (DAP-seq) technique allows the identification of transcription factors and their target genes (Huberman et al. 2021) as Jobe et al. (2021) demonstrated. This study not only confirmed that SLIM1 (sulfur limitation 1) is an essential transcription factor involved in As-resistance, but also identified targeted genes of SLIM1 in *A. thaliana* exposed to As(III). The SLIM1 mutant seedlings of *A. thaliana* were sensitive to As and accumulated more As in roots. This technique integration highlights the benefit of using novel and conventional genomic tools to gain a more thorough understanding of gene regulation under stress.

Although microarrays have provided foundational insights, they face limitations such as constrained hybridization probe capacity, background noise, and inaccurate annotations. To overcome these issues, RNA sequencing (RNA-Seq) has emerged as a more reliable alternative for detecting gene transcripts, revealing transcript variations, isoforms and low-expression genes that are often missed by microarrays (Rai et al. 2018).

This technology has proven particularly effective in studying plants under As stress, providing deeper insights into gene regulation and stress responses. For instance, Yanitch et al. (2017) used RNA-Seq to evaluate the phytoextraction efficiency of *Salix purpurea* and identified key transcripts encoding phosphate transporters (PHO1) and aquaporins (NIP1) involved in As uptake and tolerance (Yanitch et al. 2017). By using RNA-Seq, a total of 3,812 DEGs were identified in the japonica cultivar Nipponbare (*Oryza sativa* L.) shoot in As(III)-treated plants mainly related to transmembrane transportation and binding of ion and redox responses (Xiao et al. 2021). In *Panax notoginseng*, 1725 DEGs were identified using RNA-Seq, encoding hormone signaling, As hyperaccumulation with transcripts of HSF and MYB, and oxidative stress-related proteins under As stress (Liu et al. 2016). Recently, using RNA-Seq, Di et al. (2021) reported that most of the genes were downregulated in upland rice exposed to As(III) and As(V) compared to upregulated genes. The activity of antioxidative enzymes was consistent with gene expression regulating these enzymes. Despite its advantages, RNA-Seq is not without challenges. The method is resource-intensive, requiring substantial computational power to manage and analyze the large datasets it generates. Additionally, variations in RNA quality or library preparation can introduce biases, making experimental consistency critical, thus, it benefits from being integrated with complementary methods like deep sequencing for a more comprehensive understanding of transcriptomic responses (Stark et al. 2019; Ozsolak et al. 2011).

Deep sequencing further enhances RNA-Seq by directly sequencing RNA, providing an even more comprehensive view of gene regulation under stress. This approach has been used to identify novel transcripts and pathways associated with As stress. For example, Ghosh et al. (2017) reported 22 upregulated and 35 downregulated As(V)-responsive miRNAs related to growth and development, metabolic process, ROS generation and hormone signaling in *Zea mays* under As(V) stress. Using Illumina sequencing, Yu et al. (2012) reported the differential transcriptomic response of shoots and roots to As(III) stress where shoots are most sensitive to exposure time and roots are dosage sensitive. There were 59 genes related to lipid metabolism, and 324 were linked to hormones under As(III) exposure. Also, this study reported 8 upregulated genes involved in the JA signaling pathway in rice seedlings pointing towards the role of JA biosynthesis and signaling under As(III) stress. Liu and Zhang (2012) reported 67 As(III)-induced miRNAs (e.g., miR528, miR408, and miR397b), and As(III)-repressive miRNAs (e.g., miR390 and miR1316), in roots of indica rice (*Oryza sativa* L. spp. Indica). Identified RNAs were involved in oxidative stress, photosynthesis, growth, hormone signaling and heavy metal stress response by deep sequencing (miRNA Profiling). Collectively, these studies highlight the central role of miRNAs in regulating plant adaptation to As stress. By targeting genes involved in growth, metabolism, ROS scavenging, and hormonal signaling, miRNAs enable plants to prioritize stress responses over non-essential processes, which highlights the importance of miRNAs as key regulators of As tolerance. Transcriptomic studies have also revealed the importance of sulfur metabolism in As tolerance of plants as it can influence the uptake and distribution of As. The pivotal role of As(III) binding to sulfhydryl groups in glutathione (GSH) and phytochelatins (PCs) in metalloid detoxification underscores the crucial significance of sulfur metabolism for plant survival in As-contaminated soils. The synthesis of GSH and PCs, usually triggered by exposure to As, necessitates sufficient availability of the building blocks for GSH—Glu, Cys, and Gly (Finnegan and Chen 2012). Using RNA-Seq, Chauhan et al. (2020) explored the crosstalk between As toxicity and Se supplementation. Sulfate transporters (i.e., SULTR3;1 and SULTR3;6) were highly expressed in As + Se treated plants compared to As-treated plants. These transporters assimilate sulfur into cysteine which acts as a primary source for the production of GSH and PCs to display As tolerance in rice roots and shoots as observed by Chauhan et al. (2020). In addition to this, several As-transporter genes and antioxidative stress-related genes were also upregulated in As + Se treated plants. Sharma et al. (2015) also confirmed the role of sulfur metabolism during As stress and reported the induction of low-affinity sulfate transporter (SULTR2;1, Os03g09940) and sulfate transporter 2.1 (Os03g09930) negatively regulated by miR395 upon As(V) stress in rice by using miRNA Array. Transcriptomic analysis has revealed that As(III) induces sulfate transporters in both *Brassica juncea* and rice plants (Chakrabarty et al. 2009). Using microarray, Norton et al. (2008) also reported the upregulation of up to 5 sulfate transporters in rice roots upon As(V) exposure. However, for biosynthesis of Cys leading to GSH and PCs requires the reduction of sulfate to sulfide via sulfite (Takahashi et al. 2011), catalyzed by 5′-adenylylsulfate reductase. A study reported upregulation of the transcript of the 5′-adenylylsulfate reductase gene in *Arabidopsis* under As(V) stress (Abercrombie et al. 2008). Using DAP-seq, ten putative As-induced SLIM1 targeted genes were identified in *A. thaliana*, out of which nine of these genes were linked to sulfur uptake specifically GGCT2;1 which plays a role in glutathione recycling (Jobe et al. 2021).

Although genomics tools have enhanced our fundamental understanding of the genetic response of plants, changes in transcriptome at the genomic level are insufficient to mirror changes in the proteome. Various studies have reported that gene transcription does not guarantee the translation of a gene into a functional protein which can be attributed to protein–protein interactions, post-transcriptional and translational modifications, protein stability and folding (Singh et al. 2016; Wang et al. 2021; Raza et al. 2022). Thus, a detailed proteomic analysis is necessary to identify targeted proteins which could be helpful in understanding complex metabolic events.

## Proteomics

Proteomics extensively studies the proteins expressed by living organisms at a specific time, playing a crucial role in deciphering all cellular pathways at the molecular level. The field of proteomics has rapidly evolved, moving from its first-generation techniques, such as two-dimensional electrophoresis-mass spectrometry (2DE-MS), to the second-generation methods like isobaric/isotopic tagging, then to the third-generation strategies including shotgun and gel/label-free approaches, and finally to the fourth generation, which includes mass western, and selected reaction monitoring/multiple reaction monitoring (SRM/MRM) approaches (Jorrin-Novo et al. 2019; Mehmood et al. 2021).

**Fig. 5:**
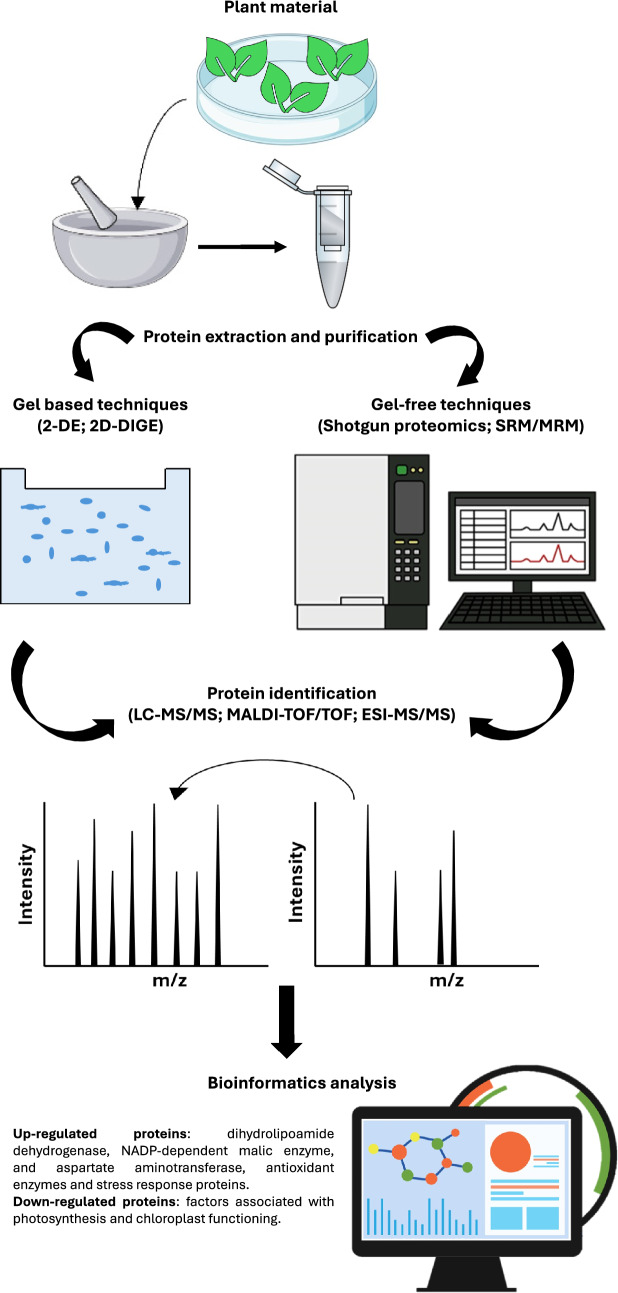
Summary outline of the different proteomics techniques used to study stress tolerance in plants. 2-DE: two-dimensional electrophoresis; 2D-DIGE: two-dimensional difference gel electrophoresis; SRM/MRM: selected reaction monitoring/multiple reaction monitoring; LC–MS/MS: liquid chromatography–mass spectrometry; MALDI-TOF/TOF: matrix-assisted laser desorption-ionization-time of flight; ESI–MS/MS: electrospray ionization-mass spectrometry

The efficiency of proteomic analysis relies on the biological sample quality used. Several components from plant tissues (i.e., phenolic compounds, lipids, polysaccharides, proteases, and secondary metabolites) can interfere with gel electrophoresis (Ahsan et al. 2009). The 2-DE is the method widely used to analyze complex protein mixtures. However, despite high reproducibility, the technique is not satisfactory for identifying and separating soluble/insoluble or highly hydrophobic core components in organelle proteomes (Ahsan et al. 2009). This problem can be resolved by using a non-gel-based multidimensional protein identification technology through the generation and separation of peptides (Liu et al. 2020). Thus, 2-DE coupled with MALDI-TOF MS (matrix-assisted laser desorption/ionization-time of flight mass spectrometry) or ESI–MS/MS (electrospray ionization tandem mass spectrometry) are the most used techniques in metal toxicity-related proteomics studies offering an overview on As stress-induced response mechanisms and factors (Fig. 5).

Both MALDI and ESI are ionization techniques widely used in MS-based proteomic analysis of plant subjected to As stress. MALDI, considered a “soft ionization” method, efficiently ionizes a broad spectrum of biomolecules, including amino acids, peptides, proteins, oligonucleotides, oligosaccharides, as well as other organic compounds like small drugs, metabolites, and large synthetic polymers. This ionization process involves the use of a matrix to absorb laser energy, resulting in the formation of ions with minimal fragmentation. It is important to note that MALDI generates ions with relatively low charges, such as singly and doubly protonated ions, facilitating straightforward molecular mass determination for most biomolecules and simplifying data interpretation compared to ESI–MS (Darie-Ion et al. 2022). It is common to combine a MALDI source with a ToF detector, often referred to as MALDI-ToF in the analysis of peptides and proteins. Chauhan et al. (2020) revealed selenium-mediated amelioration of As toxicity in rice by using 2-DE and MALDI-TOF/TOF. The results showed that selenium (Se) supplementation reversed the As-induced structural changes by suppressing As-transporting genes (NIP2;1, NIP1;1, ABCG5, NRAMP5, and TIP2;2) and sulfate transporter genes. Moreover, the upregulation of various antioxidation-related genes (GPX, GST and PRX), in As + Se treated plants, highlighted the ameliorative role of Se against As-induced oxidative stress. Similarly, 2-DE was combined with MALDI-TOF-TOF mass spectrometry to understand the underlying molecular mechanism in As toxicity and tolerance in rice (*Oryza sativa* L.). The study focused on how varying sulfur supply affects the rice leaf proteome, amino acids profile, and thiol metabolism under As(III) exposure. A total of 113 differentially expressed proteins (DEPs) were identified through 2-DE gel analysis, with 80 of these proteins successfully identified by MALDI-TOF-TOF. These proteins were primarily involved in amino acid biosynthesis, glycolysis, TCA cycle, photosynthesis, stress, and energy metabolism. Additionally, sulfur supplementation was observed to reduce As accumulation in shoots, positively influencing thiol metabolism and glycolysis towards amino acid accumulation under As(III) stress (Dixit et al. 2015). Whereas As stress seems to inhibit photosynthesis activity, the detailed proteomic response of *Artemisia annua* under As(V) concentration of 100 μM, by employing 2-DE and MALDI-TOF–MS/MS, revealed increased photosynthetic CO_2_ assimilation and usage of carbon resources. A total of 46 protein spots were observed with differential expression; these proteins were associated with ROS scavenging, energy production, and secondary metabolism (Rai et al. 2014). Later, Kumari and Pandey-Rai (2018) employed 2-DE with MALDI-TOF–MS analysis to explore the impact of salicylic acid (SA) on *A. annua* under As stress and suggested the synergistic effect of SA in modulating the plant’s tolerance to As stress. Also using the same approach, recently, it was found that DNA repair/toleration (DRT) mechanism-controlling proteins (PdDRT102) were highly upregulated in arsenate-tolerant *Populus deltoides* cv. ‘Zhonglin 2025’, which was validated by lower ROS and malondialdehyde (MDA) accumulation, structural and morphological injury, and stronger ROS scavenging ability and photosynthesis (Liu et al. 2017). However, the effectiveness of this approach depends on choosing the appropriate mass range. Nonetheless, a significant limitation arises when several proteins share the same mass-to-charge ratio (m/z), complicating the accurate identification of individual proteins (Liébana-Martos 2018). To overcome this, ESI–MS/MS provides enhanced sensitivity and comprehensive peptide sequencing capabilities. In addition to ensuring accurate protein identification, this tandem technique validates and confirms findings (Neagu et al. 2022; Noor et al. 2021). For instance, Ahsan et al. (2010) conducted a detailed study to understand how rice (*Oryza sativa* L. cv. Dongjin) reacts to As(V) toxicity by combining MALDI-ToF–MS and ESI–MS/MS. Using 2-DE, more than 700 protein spots were observed with 14 (8 with increased abundance and 6 decreased) proteins having significant changes under As exposure. These proteins were further analyzed using MALDI-ToF–MS. ESI–MS/MS was used to identify protein samples that MALDI-ToF–MS failed to identify. The upregulated proteins were associated with energy metabolic pathways including dihydrolipoamide dehydrogenase, NADP-dependent malic enzyme, and aspartate aminotransferase. The downregulated proteins were mainly associated with photosynthesis and chloroplast function highlighting the alterations in energy metabolism and photosynthesis under As exposure. However, ESI–MS/MS faces limitations such as susceptibility to matrix effects, requiring extensive sample preparation to reduce ion suppression or enhancement. It struggles with baseline separation of similar analytes, relies heavily on optimized parameters, and lacks a universal method development strategy, making standardization and consistent results challenging (Phungsiangdee et al. 2023). Liquid chromatography–mass spectrometry (LC/MS) has become a prominent analytical platform for proteomics research because of its speed, selectivity, sensitivity, accuracy, and throughput. Cesaro et al. (2015) conducted a comprehensive study on *Pteris vittata*, a known As hyperaccumulator, to understand the plant’s response to As exposure at the proteomic level by employing 2-DE and LC–MS/MS to analyze protein expression in sporophytes and gametophytes. Two key enzymes, Pv2.5–8 and PvACR2 were identified, involved in reducing arsenate to a less toxic form in both sporophytes and gametophytes.

Building upon the capabilities of LC–MS, LC–Q-TOF MS/MS is a sophisticated analytical technique that combines LC with MS/MS using a quadrupole time-of-flight (Q-TOF) analyzer. Bona et al. (2011) employed this technique to identify and characterize the protein profiles of *Pteris vittata* under As exposure and inoculation with *Glomus mosseae*. The LC-Q-TOF analysis, which involved in-gel digestion of proteins with trypsin followed by nano-high-performance liquid chromatography–MS/MS (nano-HPLC–MS/MS), revealed significant changes in the expression of key proteins. Notably, glycolytic enzymes such as glyceraldehyde-3-phosphate dehydrogenase (GAPDH) and enolase were upregulated in response to As, suggesting their involvement in enhancing glycolytic flux, providing energy and carbon skeletons for detoxification and As metabolism. Additionally, proteins related to bioenergetics, including malate dehydrogenase and ATP synthase, showed altered expression patterns, indicating increased energy demand and a broader metabolic response to As stress and mycorrhizal symbiosis. Similarly, proteins involved in nitrogen assimilation, such as glutamine synthetase, exhibit upregulation under As stress, which may be linked to the increased demand for amino acids necessary for synthesizing stress-responsive proteins and metabolites. These findings highlight the integrated metabolic and physiological responses of *P. vittata*, particularly the synergistic benefits of mycorrhizal symbiosis in improving As tolerance.

Despite its many advantages, LC-based proteomics also comes with certain limitations. The high cost of instrumentation and operation can be prohibitive, particularly for smaller research facilities. Furthermore, the techniques generate large and complex datasets, which require advanced computational tools and expertise to analyze effectively. Consistency in sample preparation is critical to ensure reproducibility, but this can be challenging to maintain, especially in large-scale studies where sample variability may influence results. Nonetheless, LC–MS/MS and LC–Q-T-MS/MS remain indispensable tools for advancing proteomic research in plants under stress conditions.

Isobaric tags for relative and absolute quantitation (iTRAQ) is a method employed in mass spectrometry to quantitatively analyze proteins by assessing the peak intensities of reporter ions that are emitted from peptides tagged with iTRAQ during the process of MS/MS (Wiese et al. 2007). Effects of exogenous methyl jasmonate (MeJA) on As tolerance in *Brassica napus* leaves were examined at the proteomic level by iTRAQ-based proteomics analysis combined with reverse phase liquid chromatography coupled with tandem mass spectrometry (RPLC–MS/MS). Notably, proteins with antioxidant properties, such as glutathione reductase and superoxide dismutase, were upregulated, indicating MeJA’s role in mitigating As-induced oxidative stress (Farooq et al. 2018). Similarly in another study (Farooq et al. 2021), two cultivars of *Brassica napus* (ZD622 and ZS758) were exposed to high As concentration, and their reactions to As toxicity were examined using the same technique (iTRAQ Labeling coupled with RPLC–MS/MS). The analysis revealed significant changes in antioxidant enzymes and stress response proteins, indicating an enhanced defence mechanism against As toxicity. Additionally, there was a notable reduction in photosynthesis-related proteins, reflecting the impact of As on photosynthetic activity. The study also highlighted cultivar-specific responses, with cultivar ZS758 showing better tolerance to As stress compared to ZD622, as evidenced by the distinct protein expression patterns (Farooq et al. 2021). While iTRAQ offers precise quantitation and multiplexing, it can be resource-intensive and may require advanced technical expertise.

## Metabolomics

**Fig. 6:**
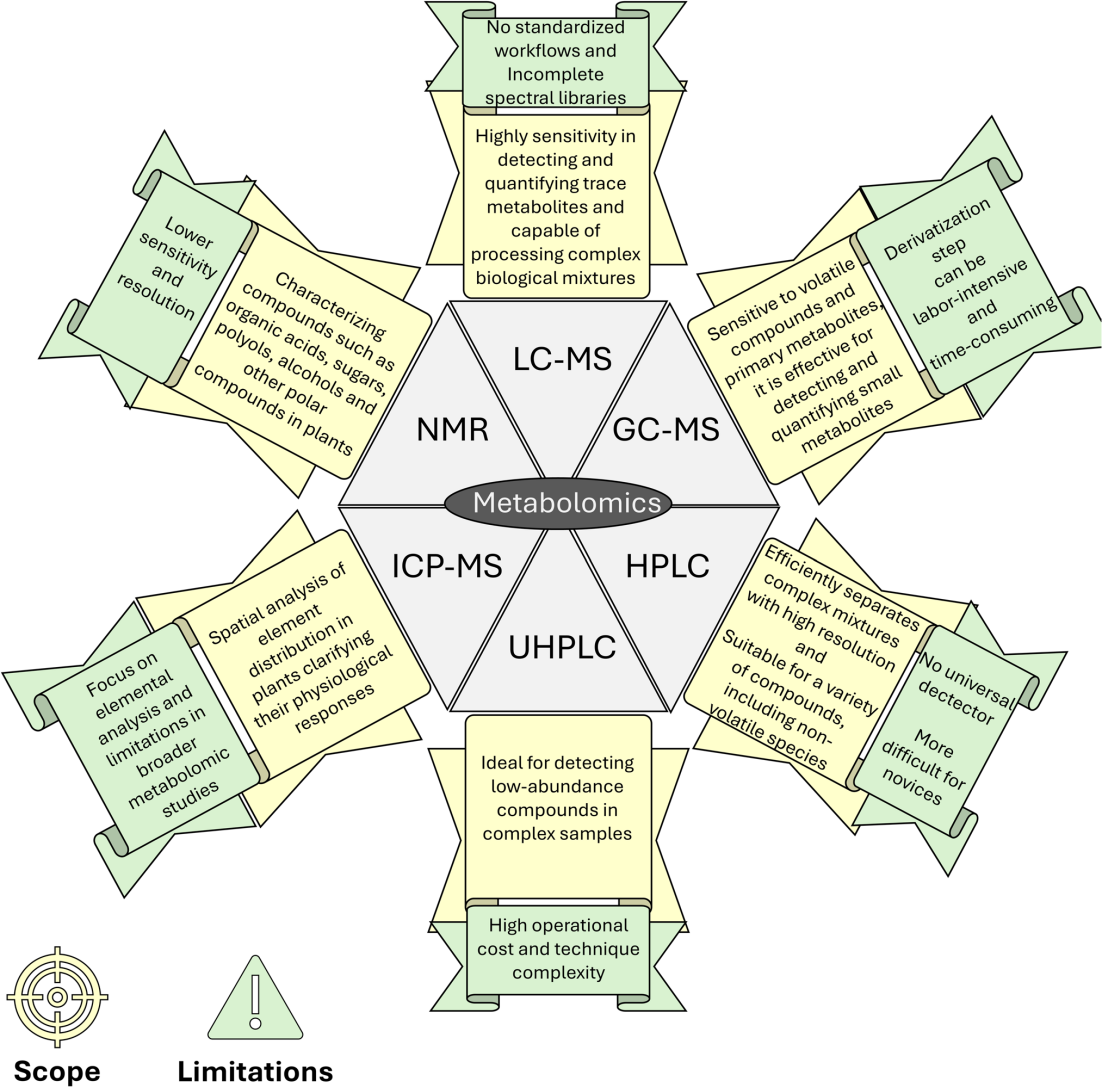
Scope and limitations of metabolomic techniques—liquid chromatography coupled with mass spectrometry (LC–MS), gas chromatography coupled with mass spectrometry (GC–MS), high-performance liquid chromatography (HPLC), ultra high-performance liquid chromatography (UHPLC), inductively coupled plasma coupled with mass spectrometry (ICP-MS), and nuclear magnetic resonance (NMR) spectroscopy—in understanding the arsenic (As) induced toxicity and tolerance in plants

Metabolomics is recognized as an emerging field that extensively identifies and measures all small molecules (less than 1 kDa), encompassing both exogenous and endogenous metabolites within living organisms. Figure 6 presents the main analytical techniques employed in metabolomics—liquid chromatography coupled with mass spectrometry (LC–MS), gas chromatography coupled with mass spectrometry (GC–MS), high-performance liquid chromatography (HPLC), ultrahigh performance liquid chromatography (UHPLC), Inductively Coupled Plasma coupled with Mass Spectrometry (ICP-MS), and nuclear magnetic resonance (NMR) spectroscopy—to understand alterations in plants under As stress (Emwas et al. 2019) along with their respective limitations. These methodologies fall into two distinct groups: untargeted metabolomics that attempts to analyze all detectable (known and unknown) metabolites from the sample (Christ et al. 2018), and, targeted metabolomics focusing on the analysis of specific metabolic categories (Roberts et al. 2012).

Untargeted metabolomics, utilizing LC–MS, is an effective method for identifying new metabolites. LC–MS serves as a multifaceted analytical tool capable of detecting and measuring various metabolites, from the small and polar to the large and non-polar, all without requiring derivatization steps. Its high sensitivity makes it particularly effective for analyzing components at the trace level (Sarkar et al. 2024; Chen et al. 2023), compiling also a qualitative profile of the substantial changes in metabolic pathways, which frequently result from stressors such as As. Indeed, unlike targeted approaches, the untargeted metabolomics captures a broad spectrum of metabolites that may not have been previously associated with stress response, enabling the identification of unexpected alterations and connections across pathways (Schrimpe-Rutledge et al. 2016). For example, Pérez-Cova et al. (2022) identified up to 100 lipids and 40 metabolites that were significantly affected by As exposure. These alterations were mostly seen in pathways associated with amino acids, glycerolipids, and glycerophospholipids, providing insights into the biochemical processes involved in As stress. Interestingly, the amount of As administered had a greater impact on the changes in the rice plants’ aerial sections than the mode of exposure (soil treatment or irrigation), highlighting the dose-dependent nature of these changes. Despite its advantages, LC–MS in untargeted metabolomics faces limitations such as matrix effects, which can lead to ion suppression or enhancement, potentially compromising data accuracy. Additionally, challenges in data interpretation due to the lack of standardized workflows and incomplete spectral libraries for metabolite identification further hinder its effectiveness (Steiner et al. 2021). Complementing LC–MS, HPLC provides an alternative resource of great analytical power that can be applied to any liquid-soluble compound that can be used as a mobile phase (Tomita et al. 2014). Das et al. (2021) conducted a study on the differential expression of two *Andrographis paniculata* genotypes when exposed to As stress and employed HPLC to measure the metabolites. The results revealed that As exposure was found to increase the production of flavonoids, such as 5,7,2′,3′-tetramethoxyflavone; significant variations were also observed in the main secondary metabolites, oxidative enzymes, and nutrient absorption, all contributing to the detoxification process under As stress. Also, by using HPLC, Tripathi et al. (2012) reported higher levels of PCs and phytochelatin synthase (PCS) activity with thiol metabolism in rice leading to As(V) tolerance and underlined the role of thiol-mediated detoxification mechanisms in conferring As tolerance. In another study, HPLC was used with the pico-tag method to analyze the amino acid profile in rice shoots under varying levels of As(III) and sulfur doses. Histidine was remarkably increased (333%) under As(III) and high sulfur supplementation, while valine significantly decreased (97%) under low sulfur supplementation with As(III) treatment (Dixit et al. 2015). Despite its advantages, HPLC lacks a universal detector, necessitating specific detectors for different analytes, which limits its versatility in multi-component analyses. Additionally, HPLC can be challenging for novices due to the complexity of its equipment and the precision required in controlling operational parameters (N’cho et al. 2016). Building on the strengths of HPLC, UHPLC delivers improved sensitivity and resolution because of smaller particle sizes and higher pressures. This can be particularly important for identifying and detecting a wider variety of chemicals, particularly those present in trace levels under metal stress, when analyzing complex plant metabolomes (Buescher et al. 2010). Yadav et al. (2021) employed UHPLC coupled with high-resolution mass spectrometry (HRMS) and identified approximately 4000 metabolites that were up- and down-regulated in rice seedlings under As stress. The altered metabolites included a range of phenolics and flavonoids known for their antioxidant properties, such as upregulated 3-hydroxybenzoic acid and genistein, and downregulated trans-cinnamic acid. Additionally, significant changes in nucleoside metabolites like inosine and hypoxanthine were observed, indicating substantial alterations in nucleotide metabolism. However, UHPLC systems are costly, up to 50% more than HPLC, and may not be compatible with existing HPLC methods. The choice of UHPLC-compatible stationary phases is limited, especially for specialized chromatography modes. Additionally, method transfer from UHPLC to HPLC is challenging due to variations in system specifications and operational conditions (Dong and Zhang 2014). While LC–MS, HPLC, and UHPLC focus on organic metabolites, Inductively Coupled Plasma Mass Spectrometry (ICPMS) provides a detailed spatial analysis of the element distribution in plants since it is not necessary to crush the sample and the ablation diameter is small enough to analyze. This aspect makes this technique more advantageous to elucidate the physiological responses of plants in terms of absorption, translocation, and storage of ions in response to metal stress (Pedrosa Diniz et al. 2019). In a study for investigating As(III) tolerance in rice (*Oryza sativa* L.), HPLC coupled with ICPMS revealed differential responses between a tolerant cultivar (Triguna) and a sensitive one (IET-4786), particularly in terms of thiol metabolism and amino acid profiles where enzymes of thiol metabolism and PCS activity were more abundant in the tolerant cultivar. Also, amino acids were significantly enhanced in the tolerant cultivar while reduced in the sensitive cultivar (Tripathi et al. 2013). In another study, reversed-phase HPLC coupled with ESI–MS was used to study metabolomic alteration in the xylem sap of *Cucumis sativus* and a few metabolites (5) were found to be altered with isoleucine to be downregulated (Uroic et al. 2012). RP-HPLC with ESI–MS is suited for a wide range of metabolites, including polar and non-polar molecules (Tang et al. 2016). The reversed-phase separation allows good retention and separation of hydrophobic molecules, whereas ESI–MS permits the ionization of polar substances. Despite its precision, ICP-MS is primarily focused on elemental analysis, limiting its scope to broader metabolomic studies.

Where LC–MS is more suitable for non-volatile compounds and secondary large metabolites, GC–MS is more sensitive to volatile compounds and primary metabolites. GC–MS is highly effective for detecting and quantifying small molecular metabolites (less than 650 Daltons) such as hydroxyl acids, alcohols, amino acids, sugars, fatty acids, sterols, catecholamines, and toxins. Often, chemical derivatization is employed to increase the volatility of these compounds, making them suitable for analysis through GC (Fiehn 2016). A detailed investigation of As(V) toxicity in *Pteris cretica* and *Spinacia oleracea* was conducted using GC–MS to identify and quantify the free amino acids in shoots, indicating a species-specific response to As stress. The results highlighted significant differences in free amino acid content and major amino acid-regulated pathways. In *P. cretica*, the total content of free amino acids (tAA), aspartate family (AspF), and glutamate family (GluF) was decreased significantly, reflecting a potential strategy for coping with As stress. Conversely, in *S. oleracea*, the content of these amino acids increased, suggesting a different metabolic adaptation to the same stress. These findings underscore the complex and varied ways plants respond to heavy metal stress at the metabolomic level (Zemanová et al. 2021). In addition to this, using GC–MS, Kanwar et al. (2015) showed that As(V) exposure induced four brassinosteroids (teasterone, castasterone, typhasterol, and 24-epibrassinolide) in *Brassica juncea* and highlighted the pivotal role of brassinosteroids in modulating the plant’s defence mechanism against heavy metal stress. The essential oil profile of *Mentha arvensis* L. by GC–MS analysis revealed the reduction in menthol content by As(V) stress highlighting the highest concentration (100 mg As·kg^−1^ of soil) of As proved toxic for mint crop (Nabi et al. 2019). Wang et al. (2011) also analyzed six organic acids (palmitic, malonic, oxalic, citric, malic, and linolenic acid) by GC–MS in two As hyperaccumulators (*Pteris vittata* and *Pteris multifida*) and a non-hyperaccumulator fern (*Pteris semipinnata*), upon As (specie not highlighted) exposure and under hydroponic conditions and concluded that organic acid concentration changes in these ferns did not directly correlate with As accumulation. However, GC–MS is limited by its requirement for volatile and thermally stable compounds, necessitating complex derivatization for non-volatile analytes. It struggles with polar metabolites, lower throughput, and peak resolution in complex mixtures. Thus, it was recommended to use GC–MS in conjunction with LC–MS to overcome its limitations and achieve a more comprehensive metabolomics profile (Zeki et al. 2020). Despite the popularity of LC–MS and GC–MS in the field of metabolomics, the utilization of NMR exhibited some advantages over other techniques including its highly automatable, non-destructive, easily quantifiable and no-biased properties with minimal or no requirement of chromatographic separation, sample preparation, or chemical derivatization. Moreover, unlike LC–MS, NMR can trace and characterize compounds such as organic acids, sugars, polyols, alcohols and other polar compounds (Guennec et al. 2014). Arora et al. (2018) confirmed the effectiveness of this method by applying it to the freshwater microalga *Scenedesmus* sp. Through the use of NMR spectroscopy, approximately 45 different metabolites were identified, including amino acids, organic acids, nucleotides, sugars, osmolytes, and phosphagens. The study concluded that the microalga coped with As toxicity by accumulating these varied metabolites. Using in vitro NMR, Delnomdedieu et al. (1994) demonstrated that the reduction of As(V) to As(III) requires two molecules of glutathione (GSH). This reduction process leads to the oxidation of GSH, forming oxidized glutathione (GSSG) by a disulfide bond. Besides these findings, the information on NMR used to understand the metabolomic response of plants under As exposure is limited (Emwas 2015).

## Conclusion and future perspectives

Omics approaches like genomics, transcriptomics, proteomics, and metabolomics have provided valuable insights into plant responses to As stress. Genomic techniques, including QTL mapping and GWAS, have identified key genetic regions and variations associated with As accumulation and tolerance in plants. These findings are crucial for breeding programs aimed at developing As-tolerant crop varieties and enhancing phytoremediation efforts. Transcriptomics (microarrays and RNA-Seq) has revealed the dynamic regulation of gene expression in response to As stress, highlighting the roles of various transcription factors/signaling pathways (WRKY, MyB, CDPK, MAPK, phytohormone and calcium signaling), and stress-responsive genes related to detoxification (Cu/Zn SOD, Mn/Fe SOD, peroxidases, GST, GR1) and transporters (e.g., phosphate, aquaporins). Proteomics (e.g., 2DE, LC–MS/MS, and iTRAQ) has identified key proteins and enzymes that play critical roles in As metabolism, such as glutamine synthetase, nitrogen metabolism, oxidative stress response, and energy metabolism. Metabolomics (e.g., LC–MS, GC–MS, and NMR) has offered a comprehensive view of the metabolic alterations, mainly involving pathways associated to flavonoids, nucleosides, amino acids, glycerolipids/phospholipids, that have been proven beneficial in identifying key biochemical processes, metabolites and/or metabolic categories related to As stress.

These high-throughput approaches reveal that plant adaptation to As exposure involves complex interactions across multiple biomolecular layers rather than changes in single biomolecule. However, constructing integrated cross-Omics networks remains a major challenge to capture these interactions. Thus, developing models and strategies to predict inter-omics connections with minimal data input and applicable for various kinds of networks represent the key step to have a holistic view of plant response to As exposure.

Looking forward, high-throughput omics platforms offer immense potential for practical applications in agriculture and environmental management. Genomics and transcriptomics can drive the development of As-resistant crop varieties through precision breeding or genome editing. Proteomics and metabolomics can identify biomarkers and key metabolic pathways that enhance phytoremediation practices, improving the efficiency of As detoxification in contaminated soils. Collaborative efforts between molecular biologists, agronomists, and bioinformatics experts are crucial to translate these insights into real-world solutions for addressing As contamination. Ultimately, advancements in omics technologies could play a pivotal role in ensuring food security and environmental sustainability in As-affected regions.

## Data Availability

Data sharing not applicable to this article as no datasets were generated or analyzed during the current study.
